# Recycling Continuous Glass Fibre-Reinforced Polyamide 6 Laminates via Compression Moulding

**DOI:** 10.3390/polym17152160

**Published:** 2025-08-07

**Authors:** Aditya Prakash Shembekar, Jason Yu, Mingfu Zhang, Chris Griffin, Dipa Ray

**Affiliations:** 1 School of Engineering, Institute for Materials and Processes, The University of Edinburgh, Sanderson Building, Robert Stevenson Road, Edinburgh EH9 3FB, UK; a.shembekar@sms.ed.ac.uk; 2 Johns Manville, 10100 West Ute Avenue, Littleton, CO 80127, USA; jason.yu@jm.com (J.Y.); mingfu.zhang@jm.com (M.Z.); chris.griffin@jm.com (C.G.)

**Keywords:** polyamide 6 (PA6)/glass fibre composites, thermomechanical properties, recycling, compression moulding, crystallinity, flexural

## Abstract

End-of-life (EoL) continuous glass fibre-reinforced polyamide 6 composites (cGF/PA6) are commonly recycled by shredding and milling, followed by injection moulding, often resulting in lower mechanical properties of second-generation products, primarily due to fibre length reduction. This study investigates the thermomechanical reprocessing of cGF/PA6 laminates via compression moulding, aiming to retain maximum mechanical performance by preserving the fibre length. Two types of 2/2 twill glass fibre-reinforced anionically polymerised polyamide 6 laminates (cGF/APA6), with either a reactive sizing agent (RS) or a non-reactive sizing agent (nRS), were reprocessed at two different temperatures, i.e., at 180 °C (between the glass transition temperature (T_g_) and the melting temperature (T_m_) of PA6) and 230 °C (above the melting temperature (T_m_) of PA6). The influence of reprocessing on matrix crystallinity, thermomechanical properties, microstructure, and flexural performance was investigated. The results revealed that reprocessing at both temperatures led to an improvement in matrix crystallinity, retention of the desirable α-crystalline phases, and an elevated T_g_ (glass transition temperature) in both reprocessed laminates. Additionally, reprocessing at 180 °C maintained the flexural performance in both, whereas reprocessing at 230 °C led to nearly 20% improvement in flexural strength for the RS laminate. The microstructural analysis of the failed flexural specimens showed matrix-coated fibre surfaces, highlighting retained fibre–matrix adhesion. Overall, the results offer insights into the potential of compression moulding as a viable alternative for recycling cGF/APA6 laminates.

## 1. Introduction

Fibre-reinforced polymer composites have emerged as stronger alternatives to conventional metals in automotive, aerospace, and defence sector applications, due to their superior strength-to-weight ratio, excellent mechanical properties, and better corrosion resistance [1,2]. These composites, typically comprising a thermoplastic or a thermosetting matrix, are reinforced with continuous or discontinuous fibre reinforcements like glass, carbon, aramid, or natural fibre [3,4]. Thermosets are more rigid than thermoplastics due to their irreversible three-dimensional crosslinked network [5,6], but the thermoplastics have a clear edge as far as their recyclability is concerned [7,8]. Additionally, their mechanical performance can be significantly improved by incorporating continuous fibre reinforcements, making them suitable candidates for high-performance applications.

Regardless of these advantages, the widespread adoption of thermoplastics was restricted due to poor fibre impregnation arising from higher melt viscosity and elevated costs associated with processing [9,10]. However, the development of ‘Reactive Thermoplastic Systems’ (RTPS), for in situ polymerisation of the thermoplastic monomers, has addressed these challenges [11] by using low viscosity monomers. For example, in the case of anionic polymerisation of APA6, the in-situ polymerisation is carried out in the presence of fibre reinforcements, using a low viscosity monomer ε-Caprolactam (3–5 mPAs), which enables effective wetting of the fibres and reduces void content. Commercially available RTPS include polyacrylate matrices from Arkema (Elium^®^), polyamides from Brüeggemann Chemical (Bruggolen^®^) [11], and polybutylene terephthalates from Metol (Metol CBT^®^) [12].

Consequently, Thermoplastic Composites (TPCs) are being adopted in various sectors, especially in the transportation sector, which demands lightweight vehicles to improve fuel efficiency and reduce carbon emissions. Currently, the transportation sector accounts for ~15% of global greenhouse gas emissions [13]. Consequently, stringent regulations, such as the EU Green Deal mandating 95% recyclability by 2025 or zero emissions from new cars by 2035 [14] and the United States Corporate Average Fuel Economy (CAFE) standards necessitating a fleet-wide average of 54.5 miles/gallon by 2025 [15] are driving the push for lightweight vehicles. In this regard, continuous glass, or carbon fibre reinforced polyamide 6 (PA6) and polyamide 66 (PA66) composites, are being explored for brake-pedals, battery enclosures, underbody shields, and door panels [16,17].

The average lifespan of a car is around 16 years [18] and prolonged exposure to a variety of environmental and mechanical stresses throughout its service life, deteriorates the performance of its End-of-Life (EoL) components and limits its potential for reuse [19]. Since the market for recycled plastics from End-of-Life Vehicles (EoLVs) is relatively scarce owing to cost constraints, much of this component waste is incinerated or landfilled [20,21]. Nearly 100 million EoLVs were predicted to have existed globally (as of 2020) [22]. The expected boom in the Electric Vehicle (EV) segment will further contribute to this environmental burden. In addition to this, the production of virgin raw materials like polyamides is highly energy-intensive and reliant on fossil fuels [23,24]. This necessitates effective recycling solutions for addressing waste management.

Various recycling techniques, including chemical, physical, mechanical, and thermal, have been explored for the recycling of PAs. Although chemical recycling techniques producing higher yields of monomeric ε-Caprolactam have been successfully developed for unfilled PA6 [25,26,27], their application has not yet been feasible for fibre-reinforced composites on a large scale due to process complexity, economic viability, and the heterogeneity of the system. Exceptionally, Toray Industries, Inc., and Honda Motor Co., Ltd. are validating a closed-loop recycling technique using subcritical water for depolymerisation of EoL GF/PA6 to ε-Caprolactam, aiming for a large-scale operation by 2027 [28]. However, such advancements are in early stages. In their review, Hirschberg et al. [23] identified mechanical recycling as the most viable recycling technique, but the quality of the end products is often compromised. Particularly for fibre-reinforced PA composites, the drop in mechanical performance was largely attributed to a reduction in fibre length during milling and injection moulding. In this regard, several studies have focused on the recycling of short glass fibre-reinforced PA6 (sGF/PA6) and PA66 (sGF/PA66) composites via injection moulding. For example, Haddar et al. [29] reprocessed GF30/PA6 pellets (30% GF by wt.%) and reported that the twice-reprocessed material retained its chemical structure compared to virgin material (validated by FTIR), but a 22.5% decline in the tensile modulus (E) and a 31.5% drop in tensile strength (TS) was observed. Likewise, Gültürk et al. [30] investigated the recycling of sGF30/PA66-based automotive waste from engine fasteners by incorporating varying ratios of regrind material in virgin composites. Although the thermal stability of the recycled material was retained, their rheology, crystallinity, and mechanical properties were altered. The drop in the mechanical performance was attributed to the reduction in fibre length below the critical fibre length. Consequently, the authors suggested that recycled composites with less than 25% recycled material and subjected to no more than two reprocessing cycles could be reutilised for automotive applications. Similarly, Pietroluongo et al. [31] examined sGF35.7/PA66 recovered from EoL automotive radiator parts and found a 34–41% drop in tensile and bending properties after three reprocessing cycles compared to the commercial virgin sGF36.2/PA66 reference. The authors proposed the reuse of these materials in automotive applications where unfilled PA66 or PA66 with lower GF content is used. Since recycling leads to degradation in product performance, researchers have also explored upcycling approaches. In this regard, Dehghani et al. [32] and Hermassi et al. [33] upcycled discarded PA6-based fishing nets with glass fibre reinforcements. Both studies reported significant improvement in tensile and impact performance with the incorporation of glass fibres.

While conventional techniques such as injection moulding are extensively employed, the use of novel approaches, like additive manufacturing, is relatively new for the recycling of GF/PA6 or GF/PA66 composites. Additive manufacturing is now being explored for the development of second-generation PA6 or PA66-based composites reinforced with recovered carbon fibres. In this regard, Wu et al. [34] repurposed discontinuous carbon fibres (reclaimed carbon fibres: rCF) by making hybrid filaments of rCF with virgin cGF and virgin PA6 powder via additive manufacturing. They observed that the additively manufactured hybrid composite (rCF/cGF-PA6, FVF 37.4%) containing an average fibre length of rCF ~500 μm, displayed a 63% higher compressive strength compared to the additively manufactured virgin cGF/PA6 composite (FVF 34.6%). Despite higher compressive strength, the tensile strength and the tensile modulus of the former were ~29% and ~17% lower than the latter. Considering these distinct advantages of both composites, the researchers fabricated a dual-composite by integrating cGF/PA6 on the tension side and rCF/cGF-PA6 on the compression side. Interestingly, the dual-composite possessed a 59% higher flexural modulus compared to the rCF/cGF-PA6 hybrid composite and a 76% higher flexural strength compared to the cGF/PA6 composite. Thus, the study successfully demonstrated the upcycling strategy for reclaimed rCFs via hybridisation and manufacturing of dual composites. Another study by Lohr et al. [35] investigated the recycling potential of the CF/PA66 filament by 3D printing. The 3D printed virgin composite was recycled twice by the same technique and the mechanical performance of the recycled composites was compared with the virgin CF/PA66. The results revealed that once recycled the composite displayed a ~20% higher flexural strength, ~20% higher flexural modulus, and a 40% higher Charpy impact strength, which was attributed to a different fibre orientation resulting from the extrusion process. However, the twice-recycled composite showed a considerably lower flexural performance (15–20%) and a ~30% lower impact strength compared to once-recycled material. This was attributed to fibre breakage and thermal degradation in the polymer during repeated processing. The authors concluded that the CF/PA66 filament can be successfully recycled once without a detrimental change in mechanical performance; however, the feasibility of subsequent recycling should be assessed based on specific applications.

Despite these attempts, limited research currently exists on the recycling of cGF/PA composites. In this context, Wilhelm et al. [36] evaluated the mechanical recycling of pultruded cGF/PA6 profiles (Fibre volume fraction (FVF) 65%) and observed that the coarse fraction composite processed by direct injection moulding (without extrusion step) retained similar (~98%) tensile strength and stiffness as compared to the virgin composite, signifying the importance of fibre length retention. Similarly, Moritzer et al. [37] explored the repurposing of cGF/PA6 composite off-cuts by injection moulding, after compounding with virgin PA6. All recycled composites displayed lower tensile and impact performance due to shorter average fibre lengths (~250–260 μm) compared to the virgin reference composite with 60 wt.% glass fibre content (~300 μm). A fully recycled composite with 64% wt.% GF content (without virgin PA6) displayed similar tensile stiffness compared to the virgin reference, but the tensile strength and impact strength were ~18% and ~45% lower, respectively.

As evident from prior studies, attrition in fibre length can be considered the principal cause for deterioration in mechanical performance. To avoid this downcycling, continuous glass fibre-reinforced anionically polymerised polyamide 6 laminates (cGF/APA6) were reprocessed via compression moulding in this research, keeping the continuity of the fibres intact. Although the scope of this research is intended for EoL composite materials, the extent of degradation during their service life can vary significantly. To eliminate this variability, this study investigates the cGF/APA6 laminates in as-received conditions. The supplied laminates were manufactured nearly four years ago and stored under ambient conditions. Additionally, reprocessing was conducted on laminates from flat-to-flat configurations via compression moulding, without altering the fibre architecture. The structural characteristics of the laminates before and after reprocessing were investigated. The crystallinity (χ) of the polymer matrix and the nature of crystalline phases were examined using the X-ray Diffraction technique (XRD). The thermomechanical response of the laminates to dynamic loading conditions was analysed by Dynamic Mechanical Analysis (DMA). The mechanical performance was evaluated using the Flexural test. Additionally, the fracture surfaces were examined by investigating the failed flexure specimens using the Scanning Electron Microscopy technique (SEM).

## 2. Experimental

### 2.1. Materials

**Figure 1:**
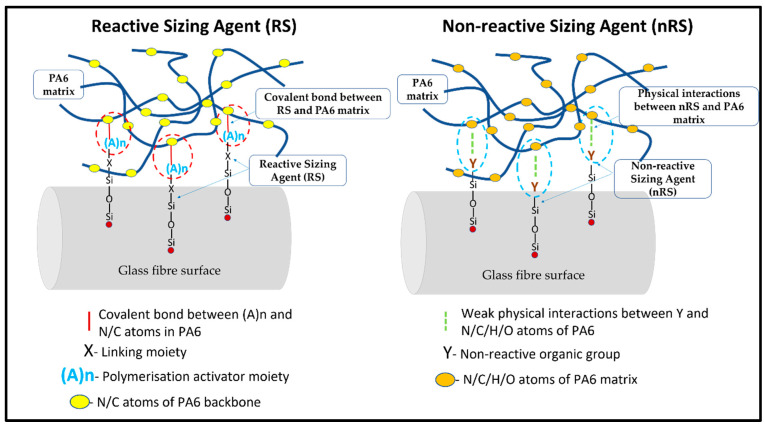
Schematic Comparison of Reactive Sizing Agent’s (RS) and Non-Reactive Sizing Agent’s (nRS) Interactions with the Matrix.

Continuous glass fibre-reinforced anionic polyamide 6 laminates with a FVF of approximately 40–44% were supplied by industrial collaborator Johns Manville (JM), (CO, USA). These laminates, also called organosheets, are manufactured by in situ anionic polymerisation of ε-Caprolactam in woven fabrics to yield an impregnated laminate with a high molecular weight PA6 matrix [38]. The laminates consist of 2/2 twill glass fabrics with a reactive (RS) and a non-reactive sizing agent (nRS), respectively. The reactive sizing agent has a “*coupling activator compound with the formula S-X-(A)n, where S represents a silicon-containing coupling moiety capable of bonding to the surface of glass fibres, X represents a linking moiety, and (A)n represents one or more polymerisation activator moieties*” [39]. The reactive sizing agent aids covalent bonding between the polymer matrix and the fibre surface, improving the fibre–matrix interfacial bonding. On the other hand, the non-reactive sizing agent is based on standard silane sizing chemistry with a general formula (RO)_3_-Si-Y where OR is an alkoxy group, Si is a Silicon atom, and Y is a non-reactive organic group which interacts with the polymer matrix via weak van der Waal forces or other physical interactions. The differences in the nature of interactions with the PA6 matrix are illustrated in Figure 1.

Throughout this study, laminates based on reactive sizing (RS) and non-reactive sizing (nRS) are referred to as cGF/APA6_RS and cGF/APA6_nRS, respectively. The terminology ‘’both laminate sets’’ refers collectively to these laminates. The technical specifications of these laminates are represented in Table 1.

To avoid batch-to-batch variations in the performance evaluation of the laminates before and after reprocessing, each reprocessed laminate had its virgin taken from the same laminate. One half of the laminate was tested as the virgin (as received), and the other was tested after reprocessing.

### 2.2. Recycling by Reprocessing via Compression Moulding

Virgin cGF/APA6_RS and Virgin cGF/APA6_nRS were reprocessed by compression moulding in a Pinette Emidecau Industries (PEI) LAB 450 P hydraulic press equipped with an integrated cooling assembly from Frigosystem chiller. The reprocessing was carried out in a 2-part cavity mould of dimensions 300 mm × 350 mm × 2 mm (Figure S1a) at two temperatures: 180 °C and 230 °C. Reprocessing details are summarised in Table 2; the compression moulding setup is shown in Figure S2.

The reprocessing parameters were chosen for the following reasons:

1. Reprocessing at 180 °C (T_g_ < Reprocessing temperature (T_RP_) < T_m_): Reprocessing above the T_g_ (45 °C −50 °C) but lower than the T_m_ (220 °C) of PA6 [40] to enable mobilisation of the amorphous segments without altering the existing crystalline domains. This would help us analyse the influence of reprocessing below the melting temperature on the χ.

2. Reprocessing at 230 °C (TRP > Tm): To melt the crystalline domains and erase residual thermal history in the laminates due to manufacturing conditions, followed by controlled cooling to enable efficient crystallisation of the molecular chains from the melt.

3. Applied pressure of 5 bar: To aid the laminate consolidation but minimise the risk of fibre misalignment in the molten state.

4. Holding time of 5 min (at TRP): To minimise the thermal degradation of the polymer matrix due to over-exposure to heat at elevated reprocessing temperatures.

The nomenclature followed for virgin and reprocessed laminates is presented in Table 3.

### 2.3. Test Methods

#### 2.3.1. Density

Each laminate’s density (*d_l_*) was measured according to ASTM D792 [41] using an OHAUS density kit (OHAUS Corporation, Parsippany, NJ, USA). At least 3 samples were tested for each laminate. An average of 3 samples is reported for every laminate.

#### 2.3.2. Fibre Volume Fraction and Void Content

The volume fraction of glass fibres (*V_cGF_*) and the void volume (*V_v_*) were measured by the matrix burn-off test according to ASTM D3171 [42]. Three samples from each laminate were held in the Nabertherm furnace (Nabertherm GmbH, Lilienthal, Germany) at 595 °C for 6 h to remove the matrix completely. The remaining mass obtained after matrix burn-off was attributed to the mass of glass fibres. Eventually, the *V_cGF_* and *V_v_* were calculated.

#### 2.3.3. X-Ray Diffraction (XRD)

XRD was used to investigate the characteristics of the crystal structure in the laminate samples. Solid specimens with dimensions of 20 mm × 18 mm × 2 mm were securely mounted onto the holder using plasticine (modelling clay) as a non-reactive adhesive medium. The analysis was conducted using the Malvern PANalytical^TM^ Empyrean X-Ray Diffractometer (Malvern Instruments, Malvern, UK) employing Co-Kα radiation (λ_Co-Kα_~1.790 Å) [43] at an operating voltage and current of 40 kV and 40 mA, respectively. The scans were conducted over a scanning range of 2θ between 10°to 40°at a 1.2°/min scanning rate. The XRD curves were deconvoluted using the Fityk software as reported in [44]. While the existing literature has predominantly reported the 2θ peak values using Cu-Kα radiation, these values were converted to match our measurements (with Co-Kα radiation) by taking into consideration the difference in X-ray wavelengths, using Bragg’s law.

(1) $$n\lambda_{Co-K\alpha }=2d\cdot Sin\theta_{1}$$

(2) $$n\lambda_{Cu-K\alpha }=2d\cdot Sin\theta_{2}$$

where

*n* = order of reflection (*n* = 1) [45]

λ_Co-Kα_ = wavelength of the Co X-ray source (1.790 Å) [43]

λ_Cu-Kα_ = wavelength of the Cu X-ray source (1.5418 Å) [46]

d = d-spacing (interplanar spacing)

θ_1_ = angle of incidence with Co X-ray source (theoretical value to be determined)

θ_2_ = angle of incidence with Cu X-ray source (available from reported literature)

The details of the conversion are given in the supplementary document.

The crystallinity was determined by calculating the ratio of the total area under the deconvoluted crystalline peaks to the total area under the XRD curve.

(3) $$\chi =\frac{\Sigma A_{c}}{\Sigma {(A}_{c}+A_{a})}\times 100$$

where

$\Sigma A_{c}$ = sum of areas under respective crystalline peaks

$\Sigma {(A}_{c}\;+\;A_{a})$ = total area under the XRD curve ($A_{a}$ area under the amorphous halo)

Similarly, the crystallite size *(L_hkl_)* perpendicular to the respective crystallographic planes was estimated using Scherrer’s equation as follows:

(4) $$L_{hkl}\;=\;\frac{K\cdot \lambda }{\beta Cos\theta }$$

where

*K* = Scherrer’s constant (0.89) [47]

λ = wavelength of the X-ray

β = Full width at half maximum of the peak

#### 2.3.4. Dynamic Mechanical Analysis (DMA)

Thermomechanical properties like the storage modulus (E′), loss modulus (E″), and the dissipation factor (tan δ) were measured using a Dynamic Mechanical Analyser (DMA 850; TA Instruments, New Castle, DE, USA). The samples were heated from room temperature to 150 °C at a heating rate of 3 °C/min and subjected to an amplitude of 20 μm along with a frequency of 1 Hz. For each set, three samples were tested.

#### 2.3.5. Flexural Testing

Flexural testing was conducted as per ISO standard 14125 [48] using a 3-point bending setup complying with Class IV specifications type. The speed of testing was set as 5 mm/min, and a span-to-depth ratio was 40:1 following the standard. Large deflection corrections, mentioned in Annex B of the standard, were taken into consideration while calculating the flexural strength.

#### 2.3.6. Microscopy

Scanning Electron Microscopy (SEM) (Hitachi TM4000 plus SEM: Hitachi High-Tech Europe GmbH, Krefeld, Germany) was used to examine the fractured surfaces of the flexure specimens. Samples were mounted onto the SEM stage using a conductive carbon tape. Observations were taken along the longitudinal cross-section of the specimen under the accelerating voltage of 15 kV.

#### 2.3.7. Statistical Analysis

The study aimed to investigate potential changes in the laminates on reprocessing; thus, a statistical *t*-test (unequal variance) was conducted to examine whether there is a significant difference in the flexural properties of the virgin and reprocessed laminates.

The equation for calculating the *t*-test value is as reported in the literature [49]:

(5) $$t=\frac{{(x}_{1}\;-\;x_{2})}{\sqrt{\frac{s_{1}^{2}}{n_{1}}\;+\;\frac{s_{2}^{2}}{n_{2}}}}\;$$

where

*t* = *t*-test value

x = sample set mean

*s* = standard deviation

*n* = number of samples

For chosen groups for comparison, higher absolute *t*-test values indicate a greater difference between them. From the *t*-test values, *p*-values are obtained from the comparison tables or in-built functions in MS Excel. If the *p*-value is less than the chosen significance level, i.e., α = 0.01 (99% confidence), the difference between the groups is statistically significant.

## 3. Results

The reprocessed laminates were examined for their densities, volume fractions of fibres, matrices, and void contents. Additionally, their crystalline morphologies, thermomechanical properties, flexural properties, and microstructural characteristics were investigated and compared against their respective virgin references.

### 3.1. Density, Fibre Volume Fractions (FVF) and Void Content

The FVF and densities of virgin cGF/APA6_RS laminates are found to be marginally higher than virgin cGF/APA6_nRS laminates, as shown in Table 4. The void content in all the virgin laminates ranged from ~5–6.5%. The reprocessing at 180 °C and 5 bar pressure did not alter the densities, FVF or the void content of both the laminate sets. The matrix remained unmelted, and the applied pressure was low; hence, the level of consolidation remained unchanged.

On the other hand, reprocessing at 230 °C caused a notable reduction in void content for both the laminate sets. The void content reduced by nearly 2% points for cGF/APA6_RS, whereas it lowered by nearly 3% points for cGF/APA6_nRS. This indicates improved consolidation of the laminates due to the molten matrix under applied pressure. The observed relatively lesser reduction in void content for cGF/APA6_RS_RP230 in comparison with cGF/APA6_nRS_RP230 might be attributed to elevated melt viscosity of the matrix arising from higher molecular weight induced by the reactive sizing agent [38,50] or stronger fibre–matrix interfacial bonding that reduced the polymer chain mobility on melting.

### 3.2. X-Ray Diffraction Analysis of Crystalline Morphology

PA6 crystallises mainly in two polymorphic forms, i.e., α and γ crystalline phases. The thermodynamically stable α-phase has a monoclinic structure with the hydrogen bonds between its anti-parallel chains, whereas the γ-crystalline phase is composed of the hydrogen bonds between its parallel chains [51]. While α-phase occurs from annealing or crystallisation at higher temperatures, the γ-phase arises on quenching [51]. Moreover, the α-phase induces strength and stiffness, whereas the latter imparts ductility in the PA6 matrix [52]. In this study, XRD analysis aimed to investigate the degree of crystallinity and existing crystalline phases in the virgin laminates and reprocessed laminates. As reported in Table 5, all virgin laminates predominantly show α-phases. 

**Figure 2:**
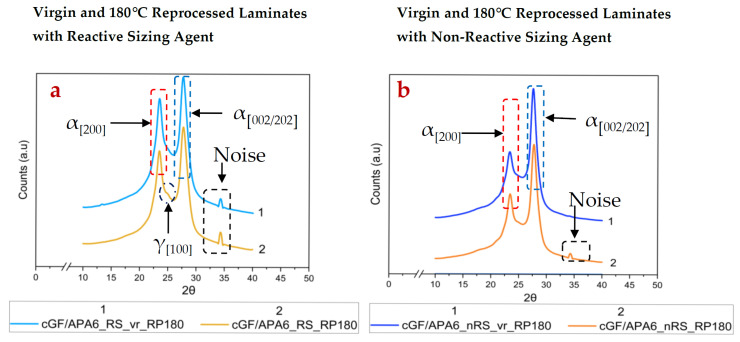
XRD curves of (**a**) virgin and 180 °C reprocessed cGF/APA6_RS laminates (**b**) virgin and 180 °C reprocessed cGF/APA6_nRS laminates. Note: Noise at 2θ = 34.2° attributed to reflection from the mounting clay (plasticine).

**Figure 3:**
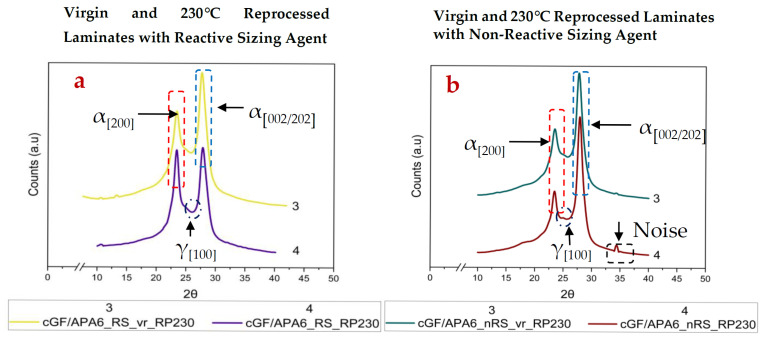
XRD curves of (**a**) virgin and 230 °C reprocessed cGF/APA6_RS laminates (**b**) virgin and 230 °C reprocessed cGF/APA6_nRS laminates. Note: Noise at 2θ = 34.2° attributed to reflection from the mounting clay (plasticine).

These observations are consistent with previously reported literature for anionically polymerised PA6 [53,54]. These α-phases occur at peak positions 2θ = 23.4° ± 0.3° [46] and 2θ = 28° ± 0.8° [46,55], attributed to the planes (200) and (002)/(202), respectively, having characteristic d-spacings 4.4 Å (±0.1 Å) and 3.7 Å (±0.1 Å) [56]. This data also aligns well with existing data with Cu-Kα radiation; however, the 2θ values have been recalculated for Co-Kα radiation (employed in this study) using Bragg’s law. As reflected in Figure 2 and Figure 3, the intensity of α_(200)_ peaks is lower than the α_(002/202)_ peaks for all laminates. This is largely dependent on the polymerisation kinetics during manufacturing. In this context, Dan et al. [54] reported that by choosing a quick/slow activator-catalyst system, and operating at higher polymerisation rates, the growth of crystals in the (200) plane can be retarded, which is likely observed in this study.

Interestingly, laminates with reactive sizing favour relatively stronger crystallisation along the (200) plane compared to laminates with non-reactive sizing, indicating that reactive bonding at the fibre–matrix interface and interfacial interactions may influence the crystalline orientation and phase development in cGF/APA6 laminates. Upon reprocessing at 180 °C, the extent of crystallinity increases for both laminate sets. The cGF/APA6_RS_RP180 showed a nearly 19% increase compared to a 9% increase for cGF/APA6_nRS_RP180 as observed in Table 5. This can be attributed to the cold crystallisation of PA6, where heating at a temperature between the T_g_ and the T_m_ may cause the Mobile Amorphous Fractions (MAF) to crystallise, whereas some portions of the Rigid Amorphous Fractions (RAF) may also convert into MAFs and further contribute to crystallisation. These observations are supported by Kolesov et al.’s findings [57]. Despite an increase in crystallinity, a reduction in average crystallite size was observed for both laminate sets, indicating that the applied temperature and holding time (at 180 °C) are insufficient to promote the growth of larger crystals from the MAFs. A minor γ-phase fraction was also observed for cGF/APA6_RS_RP180 (refer to Figure 2a).

On the other hand, reprocessing at 230 °C led to a pronounced increase in the extent of crystallinity and the crystallite size for both laminate sets. Notably, cGF/APA6_RS_RP230 showed ~23% increase in crystallinity, whereas cGF/APA6_nRS_RP230 showed ~14% increase in crystallinity compared to its virgin reference, respectively. At this reprocessing temperature, the polymer matrix undergoes complete melting, and subsequent slow cooling aids effective crystallisation and formation of larger crystalline domains. Interestingly, along with the dominant α-phases, a γ-phase fraction (~0.07) corresponding to the (100) plane was also detected for cGF/APA6_RS_RP230. This likely indicates that the reprocessing temperature initiated nucleation sites, but the 5 min. holding time may have been inadequate to cause complete transformation to the thermodynamically stable α-phase. In contrast, γ-phase contribution in cGF/APA6_nRS_RP230 was relatively negligible. Moreover, the force applied during compression moulding may have facilitated the transition of α → γ crystalline phases. Galeski et al. [58] have reported similar findings under the influence of compressive deformation. This suggests that, in addition to reprocessing temperature and holding time during compression moulding, the applied pressure also influences the extent of crystallinity and the nature of the crystalline phases.

For both the reprocessing temperatures, the extent of increase in crystallinity is higher for reprocessed cGF/APA6_RS laminates compared to cGF/APA6_nRS laminates. This indicates that the reactive sizing agent has a high impact on the overall crystallinity of the laminates. Earlier studies have shown that the nucleation efficiency and the extent of crystallinity can be influenced by multiple factors, including the type of reinforcing fibres, their surface profile and nucleating effects, and the type of sizing agent used [52,53]. The generation of crystalline regions at the fibre–matrix interface due to reactive bonding, aided by the reactive sizing agent, needs further investigation.

### 3.3. Dynamic Mechanical Analysis (DMA)

The changes in stiffness and damping performance of the laminates before and after reprocessing were investigated using DMA. The Storage Modulus (E′), Loss Modulus (E″), and tan δ were measured as a function of temperature under applied loading conditions. These properties are illustrated in Table 6.

For cGF/APA6_RS laminate set, the E′ (at 30 °C), E″ (at 30 °C), and tan δ peak values remained largely unchanged on reprocessing at 180 °C, but T_g_ increased by ~8 °C. This phenomenon can be attributed to the increased crystallinity of cGF/APA6_RS_RP180 where crystallites hinder the mobility of the amorphous chains, thereby contributing to an increased T_g_ [59]. The relatively stable damping behaviour after reprocessing likely indicates that the virgin laminate (cGF/APA6_RS_vr_RP180) already possessed a strong interfacial bonding due to the reactive sizing agent. On the other hand, some drops were observed in the storage modulus and loss modulus values in cGF/APA6_nRS_RP180 (~6.5% drop in E′, ~26.5% drop in E″).

**Figure 4:**
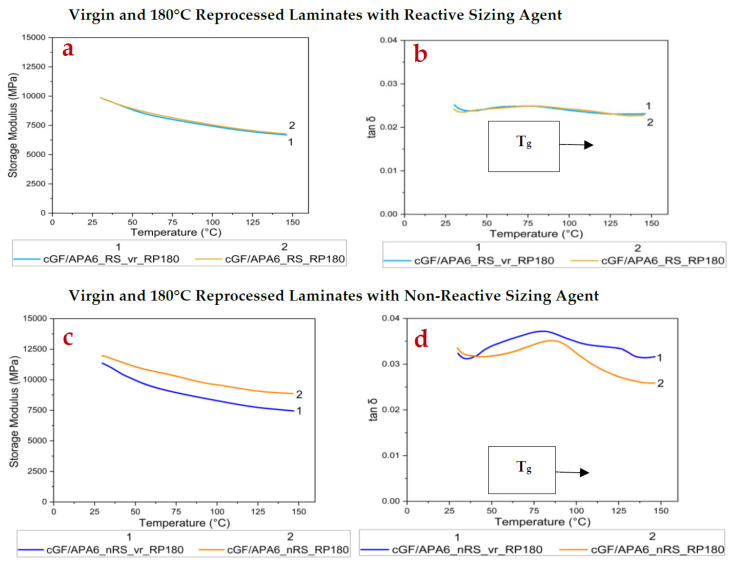
(**a**) Storage Modulus and (**b**) tan δ curves of virgin and 180 °C reprocessed cGF/APA6_RS laminates (**c**) Storage Modulus and (**d**) tan δ curves of virgin and 180 °C reprocessed cGF/APA6_nRS laminates.

The T_g_ increased by ~6.5 °C, and the tan δ peak dropped marginally compared to its virgin reference sample. These observations in cGF/APA6_nRS_RP180 (refer to Figure 4c,d) indicate decreased molecular mobility and, hence, lower damping efficiency of the polymer matrix on reprocessing.

This difference in behaviour between the laminates might be attributed to various factors induced by the reactive sizing agent during in situ polymerisation, like the extent of fibre–matrix interfacial bonding, average molecular weight, and molecular weight distribution of the in situ polymerised PA6 matrix, size, and distribution of the crystalline domains, etc.

**Figure 5:**
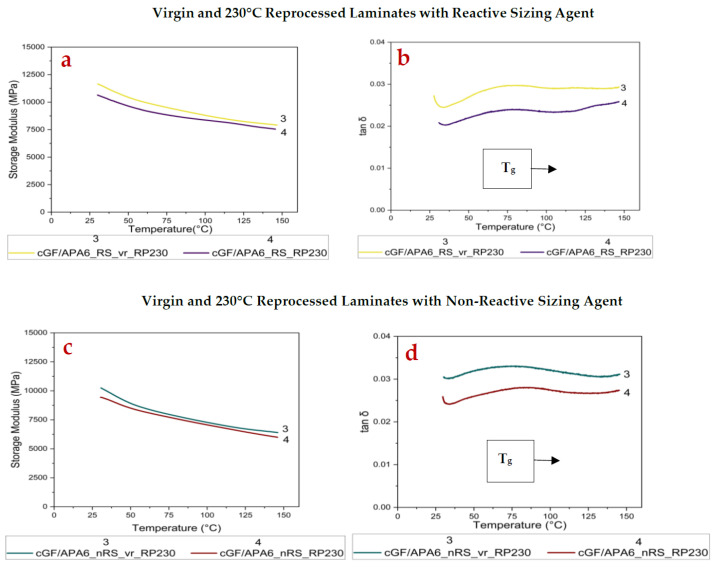
(**a**) Storage Modulus and (**b**) tan δ curves of virgin and 230 °C reprocessed cGF/APA6_RS laminates (**c**) Storage Modulus and (**d**) tan δ curves of virgin and 230 °C reprocessed cGF/APA6_nRS laminates.

On reprocessing at 230 °C, both laminates exhibited a minor drop in E′ (at 30 °C) but a considerably higher decline in E″ (at 30 °C) and tan δ peak values. These changes are evident in Figure 5a–d.

The reduction in E″ (at 30 °C) for cGF/APA6_RS_RP230 was ~30%, whereas for cGF/APA6_nRS_RP230, it was ~17%, indicating reduced energy dissipation. These changes might result from increased crystallinity or possible alterations at the fibre–matrix interface induced during reprocessing over the melting temperature, particularly in cGF/APA6_RS_RP230, due to the presence of the reactive sizing agent. Interestingly, the drop in tan δ peak value (refer to Table 6) was more pronounced for cGF/APA6_RS_RP230 compared to cGF/APA6_nRS_RP230, indicating restricted mobility of the matrix near the interface, likely due to stronger interfacial bonding facilitated by the reactive sizing agent during melt-consolidation.

### 3.4. Influence of Reprocessing on Flexural Properties

The flexural properties of virgin and reprocessed laminates were investigated, and a statistical assessment of observed results was carried out using a two-tailed *t*-test at α = 0.01 (99% confidence interval).

Reprocessing at 180 °C shows a negligible influence on the flexural properties of the reprocessed laminates. The *t*-test analysis statistically validates these observations. As presented in Table 7, the *p*-values for flexural strength and flexural modulus between the virgin and reprocessed samples (of both laminate sets) are higher than the chosen α, i.e., α = 0.01, thus changes are insignificant. While the strength and stiffness of the laminate are primarily governed by reinforcing fibres, the crystallinity and crystallite size also play a crucial role in determining the matrix strength [60,61]. These parameters ultimately influence the mechanical strength of the laminates, especially the matrix-dominated properties. As indicated by the data in Table 5, although crystallinity increased after reprocessing at 180 °C, the crystallite size reduced. These counteracting effects may have contributed to similar flexural strength for virgin and 180 °C reprocessed laminates. The matrix remains unmelted and the fibre architecture is undisturbed in this case, leading to similar flexural properties.

**Figure 6:**
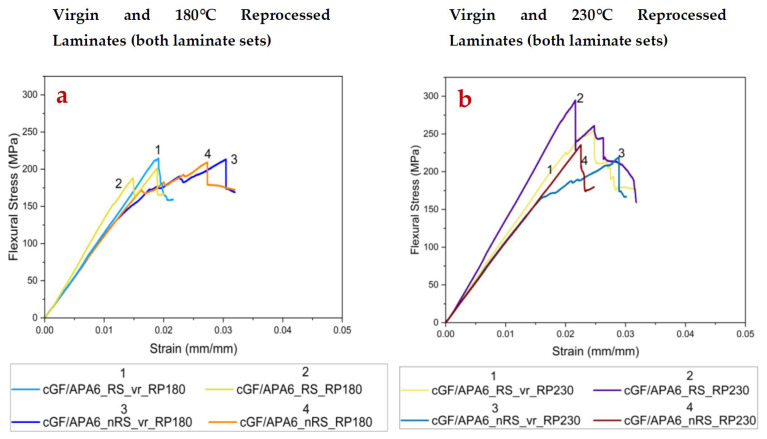
Representative Stress–Strain curves of (**a**) virgin and 180 °C reprocessed laminates (**b**) virgin and 230 °C reprocessed laminates.

In contrast to this, reprocessing at 230 °C led to a pronounced increase (~20%) in flexural strength for cGF/APA6_RS_RP230 compared to nearly 6% for cGF/APA6_nRS_RP230 despite having relatively similar void contents (~3–3.5%). This increase in flexural strength for cGF/APA6_RS_RP230 is statistically significant with *p*-value = 0.0085 < α = 0.01, likely attributed to enhanced crystallinity, larger crystallite size, and improved fibre–matrix interfacial bonding due to enhanced interactions between the covalently bonded reactive sizing and the matrix during melt-consolidation. An improved interfacial bonding facilitates efficient stress transfer under flexural loading, also highlighting the influence of the reactive sizing agent. These observations align with Fang et al. [62], who reported that interfacial modifications, such as grafting Polyetherimine (PEI)-functionalised carboxylic multi-walled carbon nanotubes (CNT) onto the glass fibre surface, promoted the formation of transcrystalline regions and enhanced mechanical performance in PEI-CNT-GF/PA6 compared to raw GF/PA6, owing to improved interfacial bonding. On the other hand, only a minor increase in flexural strength is seen in cGF/APA6_nRS_RP230 (with nRS) due to weaker physical interactions between the matrix and non-reactive sizing agent. Furthermore, the retention of flexural modulus in reprocessed laminates, supported by *p*-values > α = 0.01, indicates that melt consolidation at 230 °C did not cause undesirable fibre misalignment. The representative stress–strain curves of virgin and reprocessed laminates are illustrated in Figure 6.

### 3.5. Microstructural Analysis of Fractured Flexure Specimens

**Figure 7:**
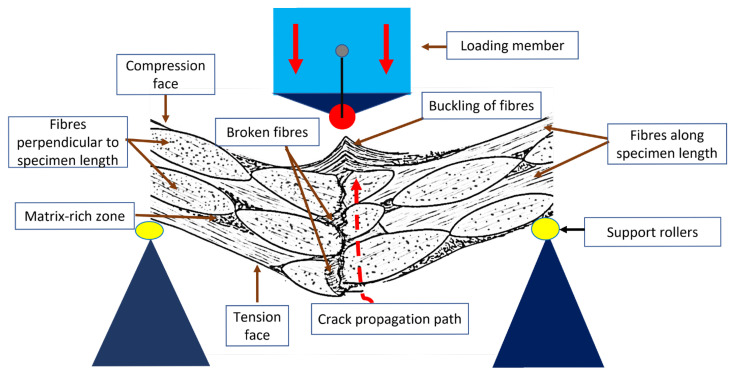
Schematic of visual observations of failed flexure specimen (longitudinal cross-sectional view).

SEM analysis of the flexural fracture surface of the specimens revealed their fracture behaviour. In all the samples, the crack initiation occurred near the tensile face and propagated through the laminate thickness towards the compressive face, as shown in Figure 7. The evidence of fibre buckling and kinking near the compressive faces indicates deformation due to compressive stresses (Refer to Figures S5 and S6).

**Figure 8:**
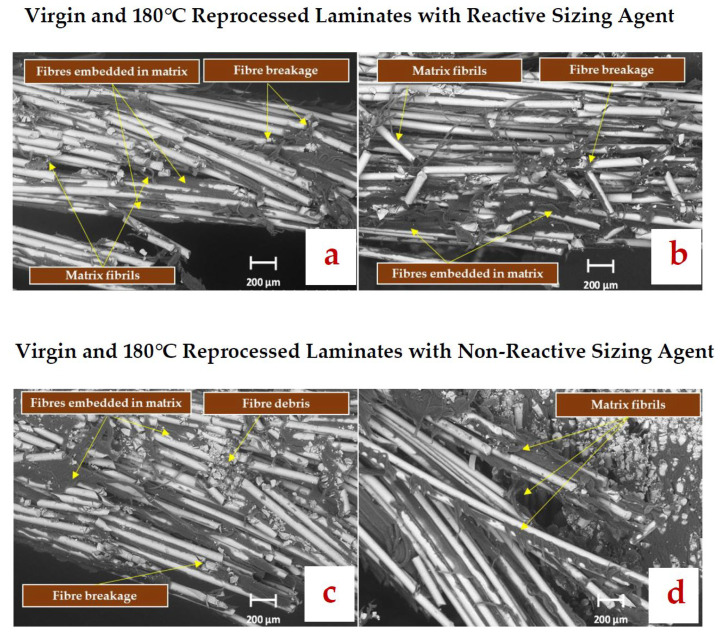
SEM micrographs of fractured flexure specimens near the tensile face at ×500 magnification for (**a**) cGF/APA6_RS_vr_RP180 (**b**) cGF/APA6_RS_RP180 (**c**) cGF/APA6_nRS_vr_RP180 (**d**) cGF/APA6_nRS_RP180.

Figure 8 shows that fibres are well-embedded in the matrix for all the laminates, with visible ductile deformation of the matrix. Moreover, a few sites of fibre breakage with attached matrix are observed. These features indicate good inter facial adhesion.

Overall, these SEM observations suggest that reprocessing at 180 °C does not significantly induce detrimental changes in the interfacial adhesion.

**Figure 9:**
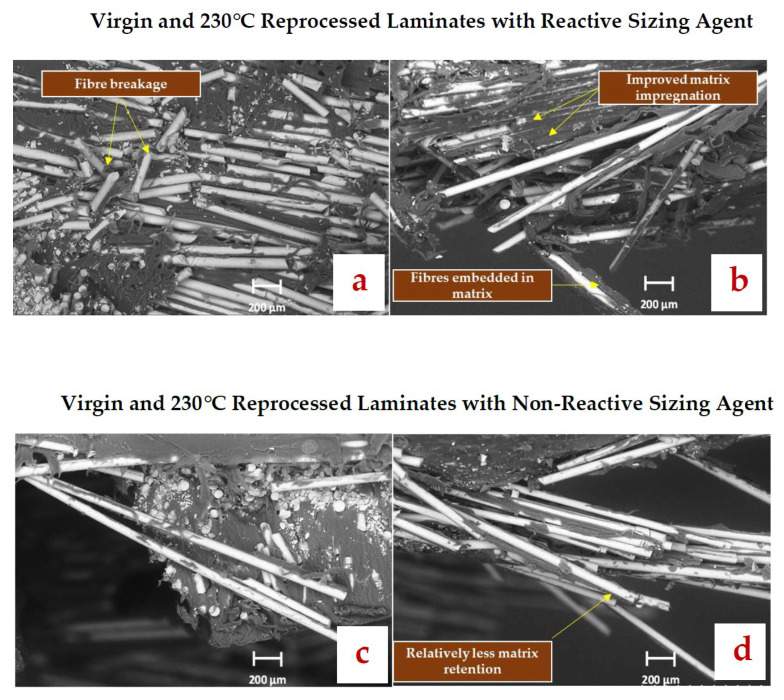
SEM micrographs of fractured flexure specimens near the tensile face at ×500 magnification for (**a**) cGF/APA6_RS_vr_RP230 (**b**) cGF/APA6_RS_RP230 (**c**) cGF/APA6_nRS_vr_RP230 (**d**) cGF/APA6_nRS_RP230.

In contrast, reprocessing at 230 °C resulted in more pronounced differences in interfacial characteristics of both laminates. Interestingly, for cGF/APA6_RS_RP230 (Figure 9b), improved fibre impregnation is evident by intense matrix coating on the fibres, indicating enhanced interfacial adhesion during melt consolidation. Although matrix residues can also be observed for cGF/APA6_nRS_RP230 (see Figure 9d), the extent of matrix retention appears to be relatively less compared to cGF/APA6_RS_RP230. Despite this, matrix fibrils are visible for all samples, indicating that reprocessing at 230 °C does not cause any matrix embrittlement. These observations suggest that 230 °C reprocessing does not deteriorate the interfacial adhesion in either laminate set. Moreover, the fibre–matrix adhesion could have potentially improved in the 230 °C reprocessed laminate with the reactive sizing agent due to enhanced covalent bonding interactions at the interface.

## 4. Conclusions

The study demonstrated the potential strategy of reprocessing two types of cGF/APA6 laminates possessing a reactive and a non-reactive sizing agent by compression moulding, and the following are the outcomes:

1. Reprocessing via compression moulding successfully preserved the fibre length and fibre architecture of cGF/APA6 laminates when reprocessing was carried out at 180 °C and 230 °C at 5 bar pressure.

2. On reprocessing at both temperatures (180 °C and 230 °C), the desirable α-crystalline phase was preserved, and the extent of crystallinity (χ) increased for both laminate sets.

For the RS laminate set, an increase in χ was more pronounced with ~19% improvement at 180 °C (for cGF/APA6_RS_RP180) and ~23% increase at 230 °C (for cGF/APA6_RS_RP230).

For the nRS laminate set, reprocessing at 180 °C and 230 °C led to an increase in ~9% and ~14% increase in χ, respectively.

Thus, the reactive sizing agent plays a crucial role in improving the crystallinity of APA6 during reprocessing.

3. All reprocessed laminates showed a higher T_g_ (from tan δ peak value), correlating well with increasing crystallinity.

4. A drop in tan δ peak values was observed for laminates reprocessed at 230 °C. However, a relatively larger drop in tan δ peak value for cGF/APA6_RS_RP230 compared to cGF/APA6_nRS_RP230 indicates more restricted matrix mobility influenced by the reactive sizing agent.

5. Reprocessing at 180 °C maintained the flexural performance of both laminate sets. On the other hand, reprocessing at 230 °C resulted in a ~20% higher flexural strength in the case of cGF/APA6_RS_RP230, likely due to improved fibre–matrix adhesion, highlighting the role of the reactive sizing agent.

6. Although 180 °C reprocessing can be an energy-efficient option, reshaping complex shapes may cause undesirable damage to fibres, limiting its potential application only to flat configurations. On the other hand, reprocessing at 230 °C may be more suitable for reshaping complex shapes, making it more viable for industrial applications.

Overall, reprocessing via compression moulding could be a viable alternative for recycling cGF/APA6 laminates on an industrial scale operation. The findings of this study may be applicable to future research on the recycling of thermoplastic composites by compression moulding or thermoforming.

## Figures and Tables

**Table 1:** Specifications of the virgin laminates (virgin: as received from the manufacturer).

Specifications	Laminate	
	cGF/APA6_vr_RS	cGF/APA6_vr_nRS
Sizing agent	Reactive sizing agent	Non-reactive sizing agent
Weave pattern in glass fabric	2/2 twill	2/2 twill
Fibre area weight (g/m^2^)	1200	1200
Yarn/Yarn count (Tex)	2400	2400
Weight rate (longitudinal/transverse) %	50/50	50/50
Thickness (mm)	2 ± 0.2	2 ± 0.2

**Table 2:** Reprocessing cycles.

Step	Reprocessing at 180 °C	Reprocessing at 230 °C
Step 1	Heating from 25 °C to 180 °C at a rate of 5 °C/min and at 5 bar pressure	Heating from 25 °C to 230 °C at a rate of 5 °C/min and at 5 bar pressure
Step 2	Holding at 180 °C for 5 min at 5 bar pressure	Holding at 230 °C for 5 min at 5 bar pressure
Step 3	Cooling from 180 °C to 25 °C at a rate of 5 °C/min and at 5 bar pressure	Cooling from 230 °C to 25 °C at a rate of 5 °C/min and at 5 bar pressure

**Table 3:** Followed Nomenclature.

Laminate Code	Woven Fabric Based on Roving	Processing Condition		Laminate Description
		Virgin	Reprocessed	
cGF/APA6_RS_vr_RP180	Reactive sizing	✅	-	Virgin reference for cGF/APA6_RS_RP180
cGF/APA6_RS_RP180	Reactive sizing	-	✅	cGF/APA6_RS reprocessed at 180 °C
cGF/APA6_RS_vr_RP230	Reactive sizing	✅	-	Virgin reference for cGF/APA6_RS_RP230
cGF/APA6_RS_RP230	Reactive sizing	-	✅	cGF/APA6_RS reprocessed at 230 °C
cGF/APA6_nRS_vr_RP180	Non-reactive sizing	✅	-	Virgin reference for cGF/APA6_nRS_RP180
cGF/APA6_nRS_RP180	Non-reactive sizing	-	✅	cGF/APA6_nRS reprocessed at 180 °C
cGF/APA6_nRS_vr_RP230	Non-reactive sizing	✅	-	Virgin reference for cGF/APA6_nRS_RP230
cGF/APA6_nRS__RP230	Non-reactive sizing	-	✅	cGF/APA6_nRS reprocessed at 230 °C

**Table 4:** Densities and volume fractions of laminates.

Laminate ID	Density *d_l_* (g/cm^3^)	Fibre (Vol.%) *V_cGF_* (%)	Matrix (Vol.%) *V_m_* (%)	Void (Vol.%) *V_v_* (%)
cGF/APA6_RS_vr_RP180	1.71	43.41	50.66	5.93
cGF/APA6_RS_RP180	1.71	42.78	52.01	5.21
cGF/APA6_nRS_vr_RP180	1.66	40.76	53.44	5.80
cGF/APA6_nRS_RP180	1.67	39.94	54.73	5.33
cGF/APA6_RS_vr_RP230	1.71	43.43	50.85	5.72
cGF/APA6_RS_RP230	1.74	43.80	52.70	3.50
cGF/APA6_nRS_vr_RP230	1.66	40.75	52.89	6.35
cGF/APA6_nRS_RP230	1.70	41.12	55.69	3.19

**Table 5:** Degree of crystallinity, nature of crystalline phases, crystalline phase fractions, and their average crystallite sizes.

Laminate ID	χ (%)	α-Phase				γ-Phase	
		α_(200)_		α_(002/202)_		γ_(100)_	
		Fraction	L*_hkl_* (Å)	Fraction	L*_hkl_* (Å)	Fraction	L*_hkl_* (Å)
cGF/APA6_RS_vr_RP180	31.25	0.38	100.8	0.62	125.3	-	-
cGF/APA6_RS_RP180	37.24	0.40	96.4	0.59	94.8	0.01	141.9
cGF/APA6_nRS_vr_RP180	30.95	0.22	78.0	0.78	101.9	-	-
cGF/APA6_nRS_RP180	33.73	0.26	66.1	0.74	97.3	-	-
cGF/APA6_RS_vr_RP230	33.74	0.38	87.9	0.62	68.1	-	-
cGF/APA6_RS_RP230	41.56	0.39	107.6	0.54	98.0	0.07	97.3
cGF/APA6_nRS_vr_RP230	31.26	0.26	98.9	0.74	103.5	-	-
cGF/APA6_nRS_RP230	35.70	0.24	91.2	0.75	105.5	0.01	173.1

Note: Minor γ-phase contributions (fractions < 0.03) are neglected in crystallinity calculations.

**Table 6:** Storage Modulus (E′), Loss Modulus (E′′), tan δ peak values, and Tg (from tan δ peak values) of virgin and reprocessed laminates.

Laminate	Average Storage Modulus at 30 °C (MPa)	Average Loss Modulus at 30 °C (MPa)	Average tan δ Peak Value	Average T_g_ from tan δ Peak Value (°C)
cGF/APA6_RS_vr_RP180	9802 ± 87	243.1 ± 26.5	0.024 ± 0.0015	71.9
cGF/APA6_RS_RP180	9796 ± 884	222.9 ± 18.0	0.023 ± 0.0015	79.7
cGF/APA6_nRS_vr_RP180	10,829 ± 416	441.7 ± 76.0	0.037 ± 0.0005	79.0
cGF/APA6_nRS_RP180	10,141 ± 2582	324.7 ± 110.1	0.033 ± 0.0026	85.7
cGF/APA6_RS_vr_RP230	11,457 ± 284	353.9 ± 86.3	0.035 ± 0.0075	79.5
cGF/APA6_RS_RP230	10,771 ± 332	244.7 ± 35.8	0.026 ± 0.0022	83.9
cGF/APA6_nRS_vr_RP230	10,021 ± 335	318.1 ± 4.1	0.033 ± 0.0008	79.5
cGF/APA6_nRS_RP230	9660 ± 350	263 ± 14.28	0.029 ± 0.0012	86.2

**Table 7:** Flexural properties and *p*-values.

Laminate ID	Average Flexural Strength (F.S.) (MPa)	*p*-Value F.S.	Average Flexural Modulus (F.M.) (GPa)	*p*-Value F.M.
cGF/APA6_RS_vr_RP180	216.7 ± 16.1	0.3001	12.21 ± 0.83	0.8244
cGF/APA6_RS_RP180	204.6 ± 18.3		12.32 ± 0.60	
cGF/APA6_nRS_vr_RP180	215.1 ± 32.5	0.9299	11.13 ± 0.34	0.2082
cGF/APA6_nRS_RP180	213.6 ± 11.8		10.87 ± 0.26	
cGF/APA6_RS_vr_RP230	255.6 ± 26.6	0.0085	12.11 ± 0.83	0.4001
cGF/APA6_RS_RP230	307.2 ± 17.5		12.48 ± 0.37	
cGF/APA6_nRS_vr_RP230	220.5 ± 15.7	0.1544	10.40 ± 0.65	0.6377
cGF/APA6_nRS_RP230	233.6 ± 9.3		10.23 ± 0.46	

## Data Availability

Data is contained within the article or Supplementary Materials.

## Supplementary Materials

The following supporting information can be downloaded at: https://www.mdpi.com/article/10.3390/polym17152160/s1. Figure S1: (a) Two-part cavity mould (2 mm cavity thickness) used for reprocessing (b) Virgin cGF/APA6_RS and Virgin cGF/APA6_nRS laminates (as received from Johns Manville); Figure S2: Schematic of compression moulding setup; Figure S3: Reprocessing at (a) 180 °C, (b) 230 °C; Figure S4: Deconvolution of an XRD curve using Fityk software (representative example: cGF/APA6_nRS_vr_RP180 sample); Figure S5: SEM micrographs of fractured flexure specimens at ×50 magnification for (a) cGF/APA6_RS_vr_RP180 (b) cGF/APA6_RS_RP180 (c) cGF/APA6_nRS_vr_RP180 (d) cGF/APA6_nRS_RP180; Figure S6: SEM micrographs of fractured flexure specimens at ×50 magnification for (a) cGF/APA6_RS_vr_RP230 (b) cGF/APA6_RS_RP230 (c) cGF/APA6_nRS_vr_RP230 (d) cGF/APA6_nRS_RP230; Figure S7: Isothermal TGA plots of (a) virgin cGF/APA6_RS (b) Virgin cGF/APA6_nRS.
