# Supplementary material for: Recycling Continuous Glass Fibre-Reinforced Polyamide 6 Laminates via Compression Moulding
Source: Polymers (Basel). 2025 Aug 7;17(15):2160. doi: 10.3390/polym17152160 (PMC12349589; doi:10.3390/polym17152160)
Supplement: Supplementary file 1 [file polymers-17-02160-s001.zip › polymers-3780917-supplementary.pdf]

Supplementary document

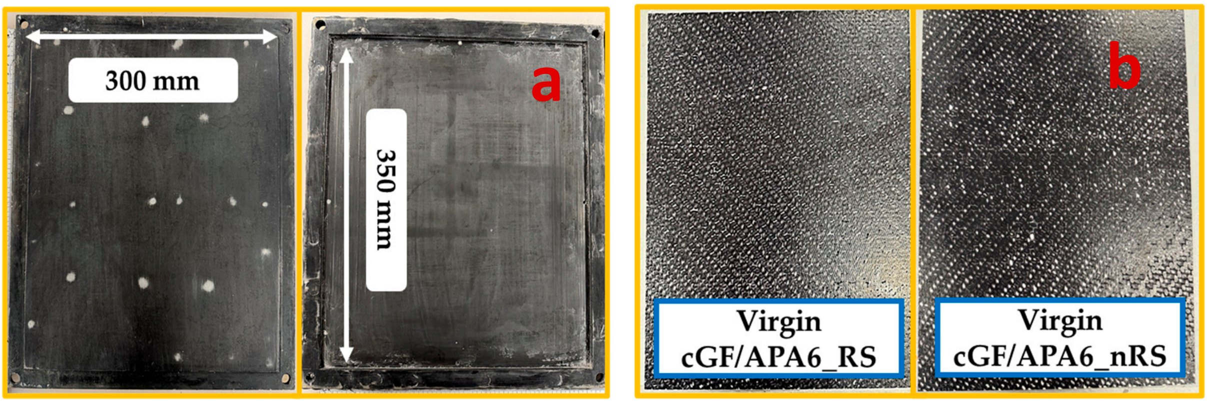

Figure S1: (a) Two-part cavity mould (2mm cavity thickness) used for reprocessing (b) Virgin cGF/APA6_RS & Virgin cGF/APA6_nRS laminates (as received from Johns Manville)

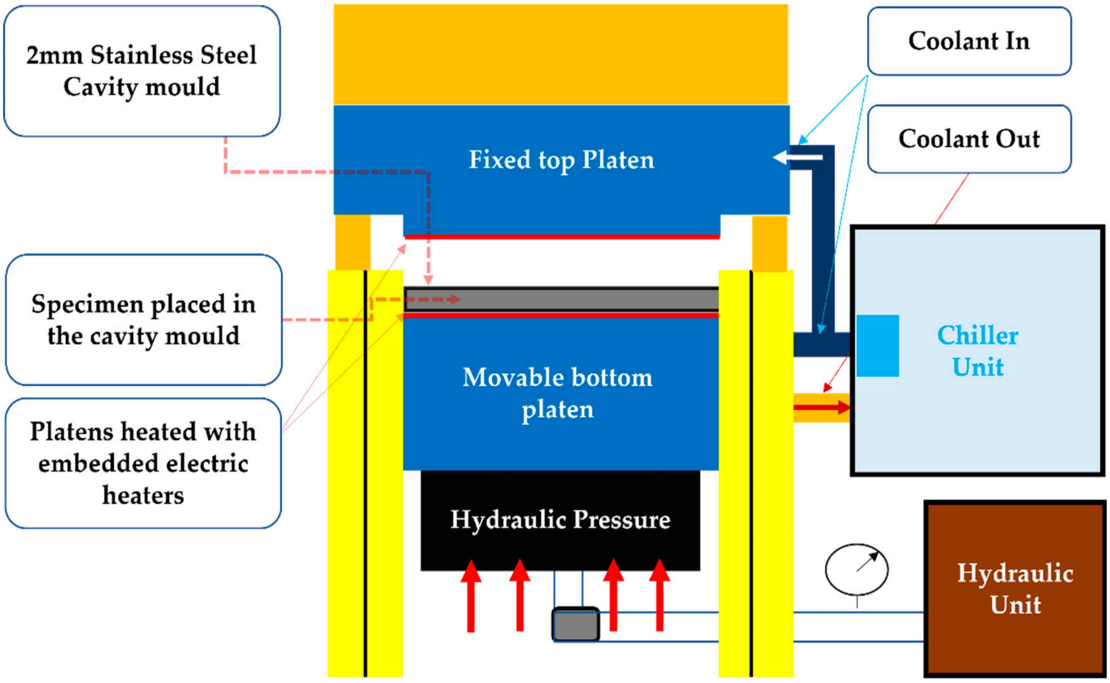

Figure S2: Schematic of compression moulding setup

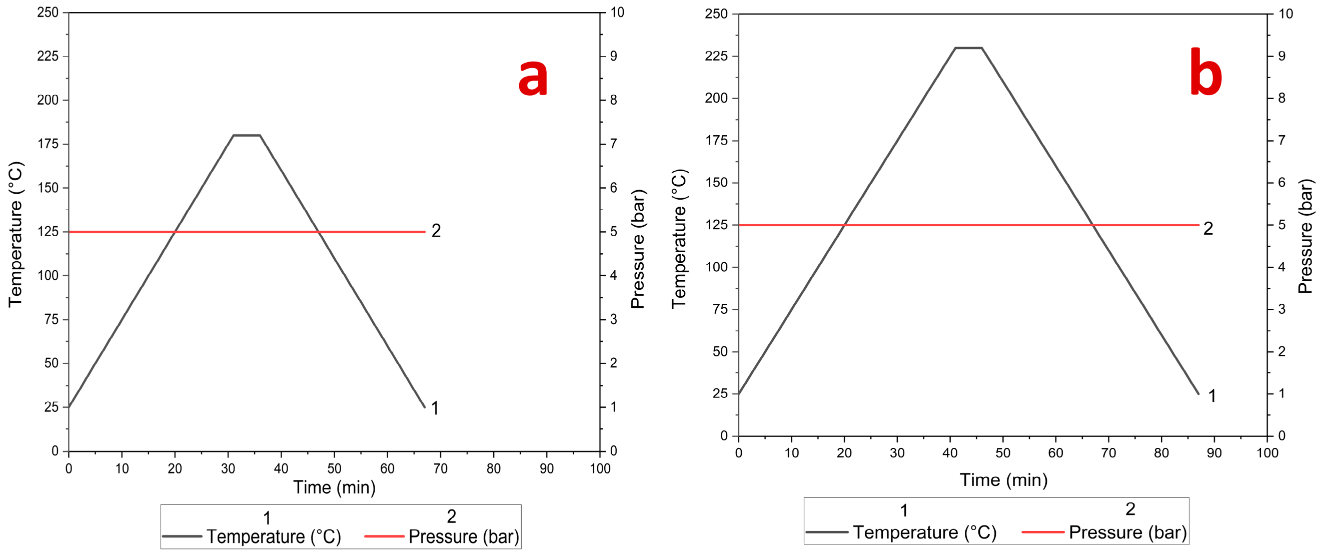

Figure S3: Reprocessing at (a) 180°C, (b) 230°C

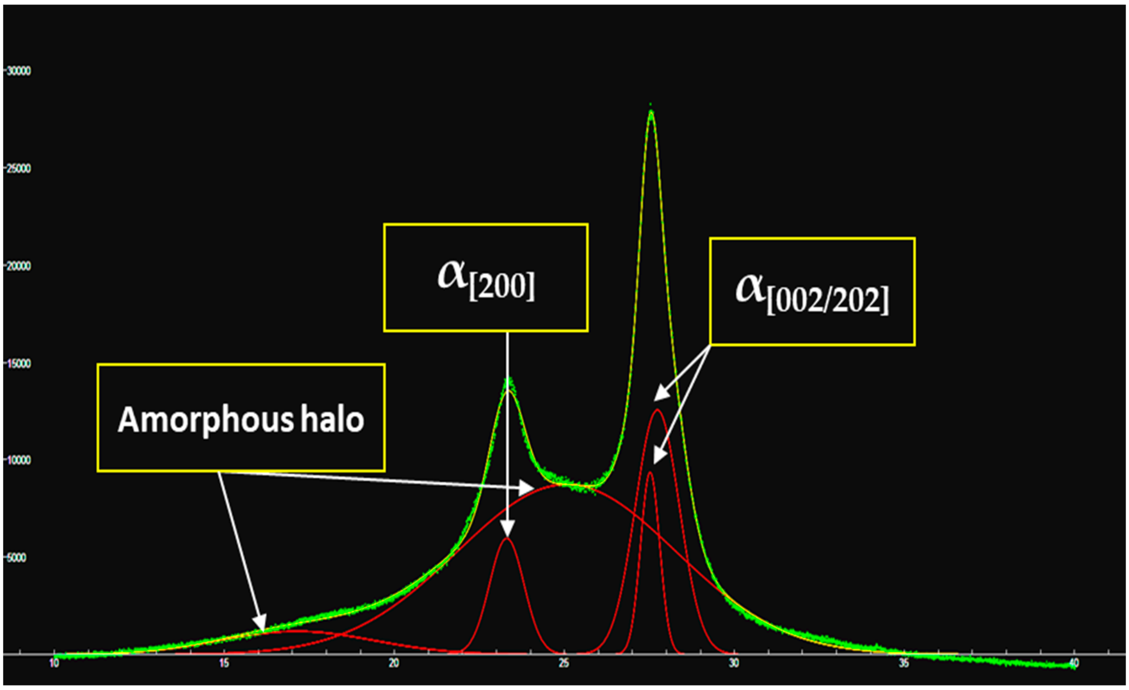

Figure S4: Deconvolution of an XRD curve using Fityk software (representative example: cGF/APA6_nRS_vr_RP180 sample)

Recalculation of theoretical 2θ values for Co-Kα radiation from existing literature based on Cu-Kα radiation. For e.g. the reported 2θ value for the $\alpha_{[200]}$ using Cu-Kα radiation in the literature is 20.01°[1]. This value can be calculated for Co-Kα radiation as follows:

$$n\lambda_{\mathrm{Co-K\alpha}} = 2\mathrm{d.\,Sin}\theta_1 \quad (1)$$
$$n\lambda_{\mathrm{Cu-K\alpha}} = 2\mathrm{d.\,Sin}\theta_2 \quad (2)$$

where:
n = order of reflection(n=1)[2]
$\lambda_{Co\text{-}K\alpha}$= wavelength of the Co X-ray source (1.790 Å)[3]
$\lambda_{Cu\text{-}K\alpha}$= wavelength of the Cu X-ray source (1.5418 Å)[1]
d= *d-spacing* (interplanar spacing)
θ_1= angle of incidence with Co X-ray source (value to be determined)
θ_2= angle of incidence with Cu X-ray source (available from reported literature)

Phase	$2\theta_2$(°)	θ_2(°)	Sinθ_2	Sinθ_1	θ_1(°)	$2\theta_1$ (°)
$\alpha_{[200]}$	20.01	10.01	0.17	0.20	11.65	23.29

Thus, the theoretical 2θ value for $\alpha_{[200]}$ crystalline phase with Co-Kα radiation is 23.29°. This value matches the obtained experimental 2θ value of 23.4°± 0.3°.

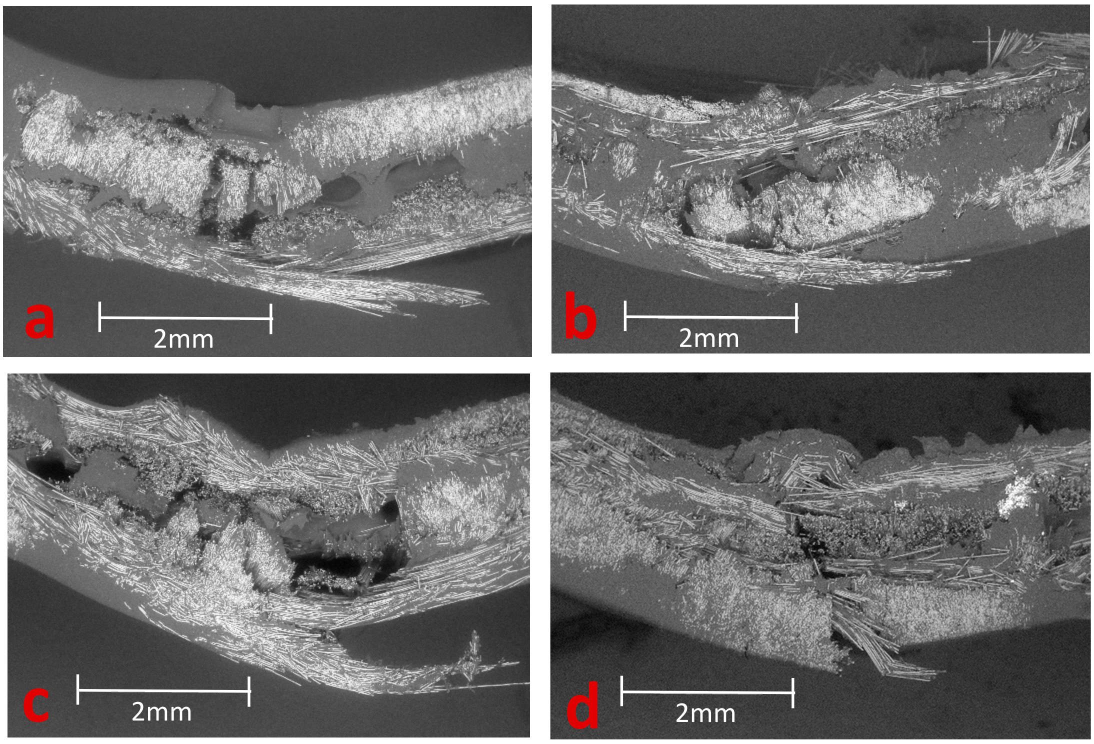

Figure S5 : SEM micrographs of fractured flexure specimens at × 50 magnification for (a) cGF/APA6_RS_vr_RP180 (b) cGF/APA6_RS_RP180 (c) cGF/APA6_nRS_vr_RP180 (d) cGF/APA6_nRS_RP180

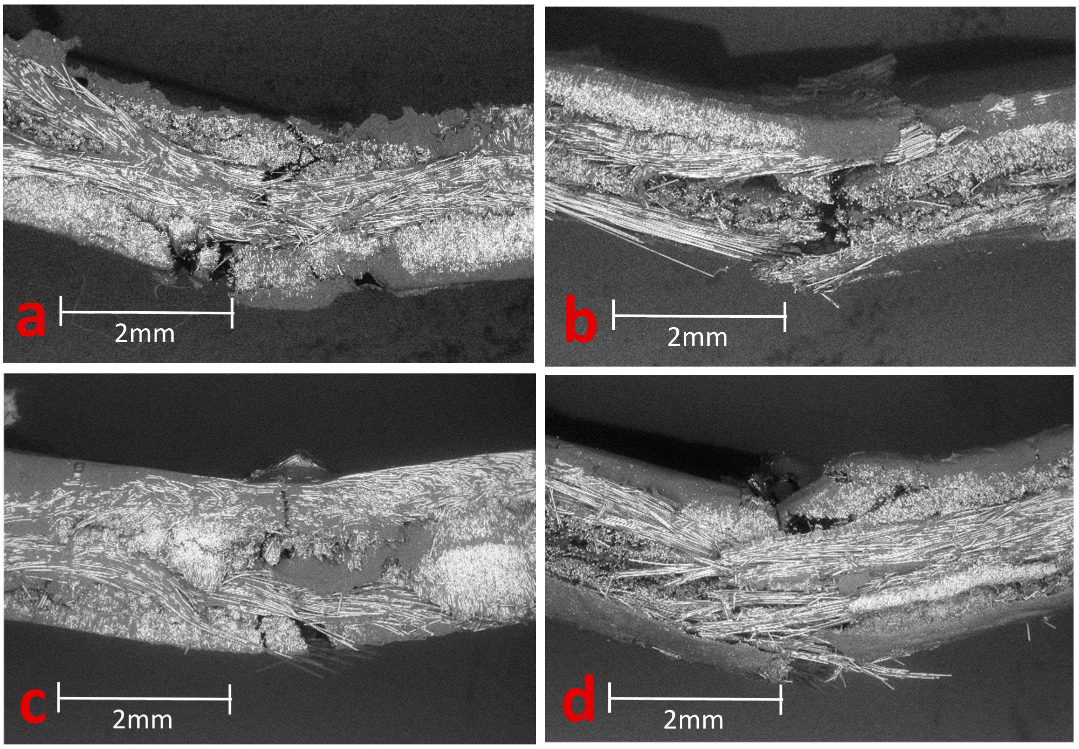

Figure S6: SEM micrographs of fractured flexure specimens at × 50 magnification for (a) cGF/APA6_RS_vr_RP230 (b) cGF/APA6_RS_RP230 (c) cGF/APA6_nRS_vr_RP230 (d) cGF/APA6_nRS_RP230

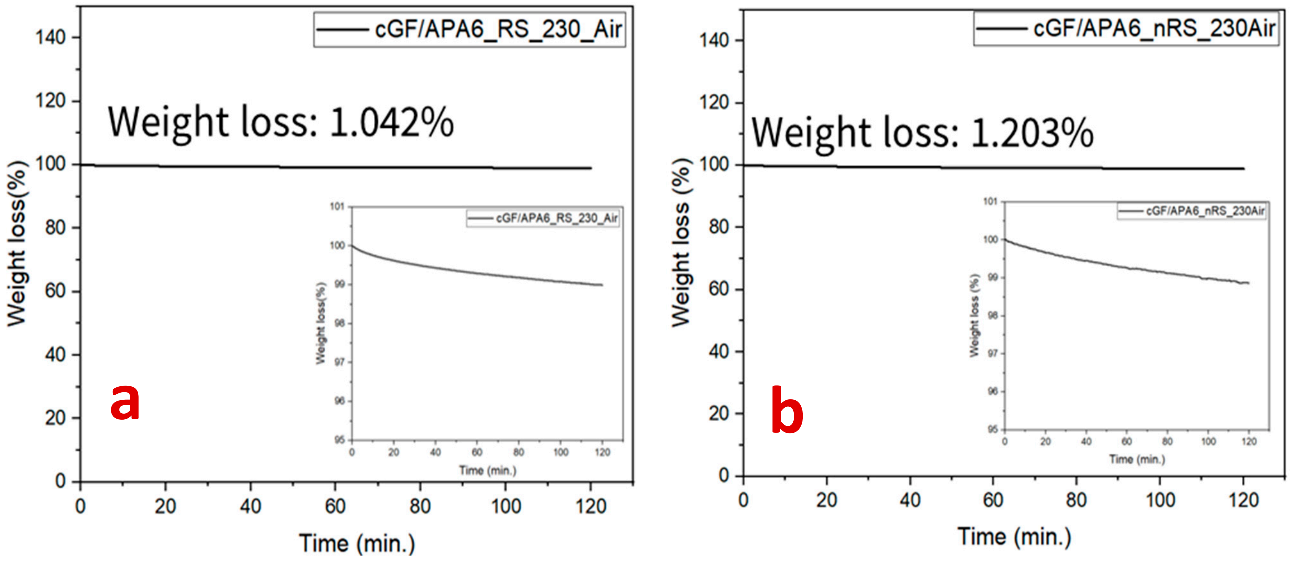

Figure S7: Isothermal TGA plots of (a) virgin cGF/APA6_RS (b) Virgin cGF/APA6_nRS

References:

[1] M. Skorupska, M. Kulczyk, P. Denis, D. Grzęda, A. Czajka, and J. Ryszkowska, “Structural Hierarchy of PA6 Macromolecules after Hydrostatic Extrusion,” *Materials*, vol. 16, no. 9, May 2023, doi: 10.3390/ma16093435.

[2] L. A. Al Juhaiman, A. A. Aljaghwani, and W. K. Mekhamer, “Preparation and Characterization of Polyamide6/Organic Clay Nanocomposite as protective coating for Carbon Steel,” *Int J Electrochem Sci*, vol. 15, no. 7, pp. 6938–6954, Jul. 2020, doi: 10.20964/2020.07.79.

[3] J. Merz, D. Cuskelly, A. Gregg, A. Studer, and P. Richardson, “On the complex synthesis reaction mechanisms of the MAB phases: High-speed in-situ neutron diffraction and ex-situ X-ray diffraction studies of MoAlB,” *Ceram Int*, vol. 49, no. 23, pp. 38789–38802, Dec. 2023, doi: 10.1016/j.ceramint.2023.09.216.
